# Floral traits driving reproductive isolation of two co-flowering taxa that share vertebrate pollinators

**DOI:** 10.1093/aobpla/plv127

**Published:** 2015-11-11

**Authors:** Joel A. Queiroz, Zelma G. M. Quirino, Isabel C. Machado

**Affiliations:** 1 Programa de Pós-Graduação em Biologia Vegetal, Departamento de Botânica, CCB, Universidade Federal de Pernambuco, 50372-970 Recife, PE, Brazil; 2 Departamento de Engenharia e Meio Ambiente, CCAE, Universidade Federal da Paraíba, Rio Tinto, PB, Brazil; 3 Departamento de Botânica, CCB, Universidade Federal de Pernambuco, 50372-970 Recife, PE, Brazil

**Keywords:** Bats, chiropterophily, generalized and specialized systems, hummingbirds, *Ipomoea*, pollinator sharing, Trochilidae

## Abstract

Floral attributes evolve in response to frequent and efficient pollinators, which are potentially important drivers of floral diversification and reproductive isolation. In this context, we asked, how do flowers evolve in a bat–hummingbird pollination system? Hence, we investigated the pollination ecology of two co-flowering *Ipomoea* taxa (*I. marcellia* and *I.* aff. *marcellia*) pollinated by bats and hummingbirds, and factors favouring reproductive isolation and pollinator sharing in these plants. To identify the most important drivers of reproductive isolation, we compared the flowers of the two *Ipomoea* taxa in terms of morphometry, anthesis and nectar production. Pollinator services were assessed using frequency of visits, fruit set and the number of seeds per fruit after visits. The studied *Ipomoea* taxa differed in corolla size and width, beginning and duration of anthesis, and nectar attributes. However, they shared the same diurnal and nocturnal visitors. The hummingbird *Heliomaster squamosus* was more frequent in *I. marcellia* (1.90 visits h^−1^) than in *I.* aff. *marcellia* (0.57 visits h^−1^), whereas glossophagine bats showed similar visit rates in both taxa (*I. marcellia*: 0.57 visits h^−1^ and *I.* aff. *marcellia*: 0.64 visits h^−1^). Bat pollination was more efficient in *I.* aff. *marcellia*, whereas pollination by hummingbirds was more efficient in *I. marcellia*. Differences in floral attributes between *Ipomoea* taxa, especially related to the anthesis period, length of floral parts and floral arrangement in the inflorescence, favour reproductive isolation from congeners through differential pollen placement on pollinators. This bat–hummingbird pollination system seems to be advantageous in the study area, where the availability of pollinators and floral resources changes considerably throughout the year, mainly as a result of rainfall seasonality. This interaction is beneficial for both sides, as it maximizes the number of potential pollen vectors for plants and resource availability for pollinators.

## Abstract (precis)

We investigated pollination in two plants that shared the same pollinators (bats and hummingbirds), overlapped in flowering and showed similarities in floral traits, favouring the mixture of pollen and the loss of plant genetic material. However, floral length differences between the plants enabled pollen deposition on different parts of the body of pollinators, reducing the mixture of pollen. In addition, differences in the opening times of the flowers can explain the more effective pollination by bats in one of the plants and by hummingbirds in the other. This trend to pollinator specialization may favour the coexistence of these plants.

## Introduction

Historically, pollination biology has considered the diversification of floral traits of angiosperms as an adaptive response to selective pressures mediated by their pollinators (Darwin 1862; Stebbins 1970). It has also been a general idea that the set of floral attributes of a species should be related to its more frequent and effective pollinator (Stebbins 1970). However, determination of the most important pollinator does not seem so easy in some systems, especially those in which the number of floral visitors of different taxa is high (Herrera 1987; Olesen *et al*. 2007; Maldonado *et al*. 2013; Avila *et al*. 2015). On the other hand, since floral features may in fact reflect a rather diverse pollination history (Waser *et al*. 1996), classifying the flowers in a given pollination syndrome can mask the importance of ‘secondary’ or ‘tertiary’ pollinators as drivers of particular floral traits.

The pollination efficiency of different floral visitors has been measured by quantitative (e.g. visits ratio) and qualitative (e.g. per-visit pollen removal and deposition, pollen germination ratio or fruit and seed set) components (Muchhala *et al*. 2009; Maldonado *et al*. 2013; Rocca and Sazima 2013; Zych *et al*. 2013; Aslan *et al*. 2014). Such efficiency components exhibit a high variation in pollination systems whose pollinators are taxonomically very close (Rocca and Sazima 2013), and even more in those systems whose pollinators belong to different functional groups or to distant taxa (Schemske and Horvitz 1984; Sazima *et al*. 1994; Martén-Rodríguez *et al*. 2009; Muchhala *et al*. 2009; Amorim *et al*. 2013; Maldonado *et al*. 2013).

Bat–hummingbird pollination systems have been interpreted as transitions from ornithophily to chiropterophily (Buzato *et al*. 1994; Sazima *et al*. 1994) or as evolutionary stable generalist systems (Muchhala *et al*. 2009). Variations in pollinator effectiveness have been demonstrated, for example, in pollination systems involving bats and hummingbirds and different plant species, such as *Siphocampylus sulfureus* (Campanulaceae) (Sazima *et al*. 1994), *Abutilon* (Malvaceae) (Buzato *et al*. 1994), *Burmeistera* (Muchhala 2003), *Aphelandra acanthus* (Acanthaceae) (Muchhala *et al*. 2009) and *Encholirium* (Bromeliaceae) (Christianini *et al*. 2013; Queiroz *et al*. 2016). Those variations in the role of pollinators can favour generalization (Waser *et al*. 1996). For example, in the case of *A. acanthus*, primary pollinators (e.g. bats) transfer a large amount of intra- and interspecific pollen to the stigma of the plant (Muchhala *et al*. 2009). This decrease in the quality component of bat pollination service makes secondary pollination (e.g. hummingbirds) beneficial and, hence, the generalized pollination system is selected (Muchhala *et al*. 2009).

From the plant's perspective, a bat–hummingbird pollination system can positively affect fitness by increasing the number of pollen vectors and insurance in seed production, even when one of the pollinator taxa becomes scarce (Muchhala 2003, J. Queiroz, unpubl. data). However, it can also negatively affect fitness, by means of pollen loss and stigma clogging by interspecific pollen deposition when a pollinator visits other plant species (Lloyd and Yates 1982; Murcia and Feinsinger 1996; Caruso and Alfaro 2000; Johnson and Steiner 2000; Morales and Traveset 2008).

The mixture and loss of pollen are common when congeneric plant species share their pollinators (Rathcke 1983; Waser 1983; Caruso and Alfaro 2000). These negative effects of pollinator sharing can be reduced if plants involved show divergences in floral structures that allow mechanical isolation (Grant 1992; Fulton and Hodges 1999) and place their pollen on different body parts of the pollinator (Kay 2006; Muchhala and Potts 2007; Muchhala 2008). They can also be reduced through floral specialization in different pollinators (Grant 1992; Christianini *et al*. 2013) via morphological restriction, which can guarantee reproductive isolation (Grant 1949). Competition through interspecific pollen transfer can drive character displacement in plant species that coexist and share the same pollinators (Muchhala and Potts 2007).

In the present study, we investigated the pollination ecology of two co-flowering taxa of *Ipomoea* (Convolvulaceae). Previous records of bat visits conducted in one of the taxa studied here, *Ipomoea* aff. *marcellia* (Z. G. M. Quirino and I. C. Machado pers. obs.), indicated that their ‘cup-shaped’ (sensu Fleming *et al*. 2009), whitish green and twilight flowers were related to chiropterophily. Although there are suggestions of bat pollination for a few species of *Ipomoea* (Butanda-Cervera *et al*. 1978; Dobat and Peikert-Holle 1985; Sánchez and Medellín 2007; Fleming *et al*. 2009), the role of bats in the pollination was tested only for *I. murucoides* (Caballero-Martínez *et al*. 2012). Here, we present two new records of bat pollination in the genus *Ipomoea* (*I. marcellia* and *I.* aff. *marcellia*). In addition to bats, floral traits of these two taxa indicate that hummingbirds are also visitors of both studied *Ipomoea.* Thus, we found a suitable model to compare the role of nocturnal and diurnal vertebrates in pollination and to test hypotheses about the mechanisms that could drive floral adaptation to particular pollinators and, consequently, reproductive isolation in similar and co-flowering species.

Our main questions were: (i) do differences in floral attributes between both *Ipomoea* taxa cause reproductive isolation through differential pollen placement on pollinators? (ii) What is the breeding system of these two *Ipomoea* taxa? and (iii) are bats more efficient pollinators that hummingbirds in both *Ipomoea* taxa? We expected that: (i) floral morphology (e.g. corolla width and length) to be the main attribute related to reproductive isolation between the two studied *Ipomoea*; (ii) higher fruit and seed per fruit set in outcrossing (intrataxa) than in self-pollination and outcrossing (intertaxa). And, given that bats have been considered more efficient than hummingbirds in some bat–hummingbird pollination system (Muchhala *et al*. 2009; Muchhala and Thomson 2010), we also expected: (iii) floral attributes primarily related to bat pollination and secondarily to hummingbird pollination in both *Ipomoea t*axa, and (iv) pollination by bats should result in a higher production of fruits and seeds than pollination by hummingbirds.

## Methods

### *Ipomoea* taxa and study area

*Ipomoea* is a plant genus with ∼650 species distributed in the tropics and subtropics (Austin 1998). Approximately 140 species of *Ipomoea* are known in Brazil (Ferreira *et al*. 2013). They can occur in several types of vegetation, from dry Caatinga shrublands to Amazon wetlands (Paz *et al*. 2013). This genus is predominantly melitophilous (Bullock *et al*. 1987; Gottsberger *et al*. 1988; Galetto and Bernardello 2004; Pick and Schlindwein 2011). There are only a few cases of pollination by birds (Machado and Sazima 1987; Galetto and Bernardello 2004) and rare cases of pollination by bats reported (Caballero-Martínez *et al*. 2012).

The two *Ipomoea* taxa selected as study models are climbers with overlapping distribution in the Almas Farm. They overlap their flowering periods, which occur at the end of the rainy season and beginning of the dry season, between July and October. The sampled individuals were distributed along 2500 m, frequently in open vegetation along trails and at the edge of rocky outcrops.

**Figure 1.**
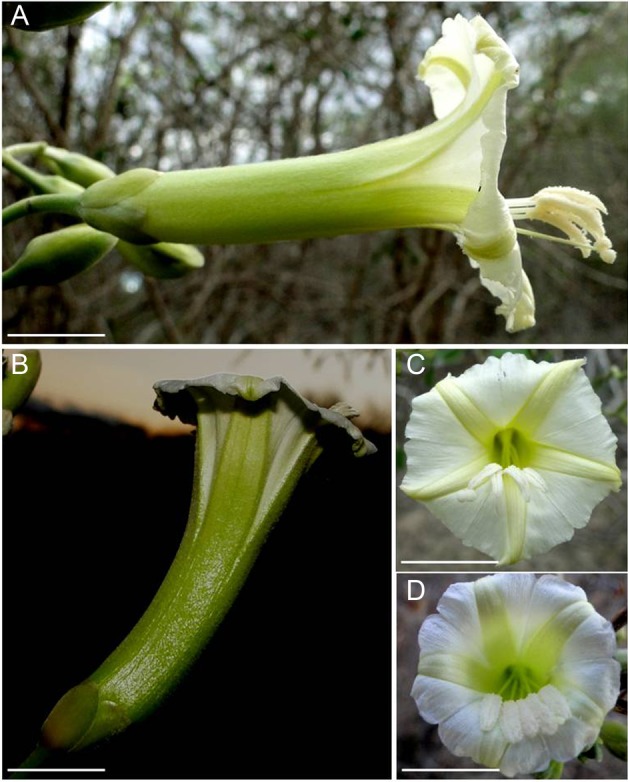
Differences in flower arrangement in two co-flowering *Ipomoea* (Convolvulaceae) in a Caatinga area, northeastern Brazil. Flowers of *I. marcellia* are arranged horizontally (A); flowers of *Ipomoea* aff. *marcellia* are arranged vertically (B); frontal view of the corolla opening and anther and stigma arrangement in *I. marcellia* (C) and *I.* aff. *marcellia* (D). Scale bars: A–D = 1 cm.

There is no information about whether both *Ipomoea* studied in this work, have overlapping distribution in other areas, considering that only one of the two taxa appears in floristic lists, identified as *I. marcellia* (Buril *et al*. 2013). The other taxon has not been described yet, or has been misidentified as *I. marcellia*, and could be new to science. Here, we denominated it as *Ipomoea* aff*. marcellia*, due to its similarity to *I. marcellia*. Several differences between these two plants, both in floral morphology and pollination ecology, which will be assessed in the present study, justified considering them as two separate taxa. The flowers of both *Ipomoea* taxa are ‘cup-shaped’ (sensu Fleming *et al*. 2009), whitish green and have a tubular corolla. They are horizontally arranged in *I. marcellia*, and in an upward, almost vertical position in *Ipomoea* aff. *marcellia*, due to a curvature of the corolla (Fig. 1).

Individuals of *I. marcellia* occur close to the ground, and have been frequently observed climbing on shrubs of 1.5 and 2.0 m height. In turn, *I.* aff *marcellia* shows high variation in height among the species it climbs on, from shrubs close to the ground to trees (4.0–6.0 m). We did not observe individuals of *I. marcellia* in supports as high as individuals of *I.* aff. *marcellia*.

We developed the field study in Almas Farm (7°28′45″S and 36°54′18″W), a 3505 ha private reserve located in the Brazilian Caatinga. This area has the lowest rainfall indices in Brazil (600 mm year^−1^) and strong rainfall seasonality (Prado 2003). It has two marked seasons: a rainy season, concentrated in the first 3 months of the year and a dry season that lasts between 6 and 9 months. A shrubby-arboreal, thorny, deciduous vegetation is typical of the hyperxerophilic caatinga (Rodal and Sampaio 2002).

### Morphometry and floral anthesis

Field activities were carried out from July to October 2012, covering the entire flowering period of the studied *Ipomoea* taxa. To investigate differences in floral attributes between the two *Ipomoea* taxa, we collected data on length and diameter of the corolla tube, and length of the style and pistil. To guarantee inter-individual floral variability, we measured a single flower per individual (*n* = 20 individuals per taxon). We monitored anthesis in different individuals (*n* = 20 flowers per taxon) and recorded the time of opening of flower buds, anther dehiscence, stigmatic receptivity and senescence of flowers. To test for stigmatic receptivity, one flower per individual (*n* = 15 individuals per taxon) had the pistil dipped in hydrogen peroxide, and the stigma was considered receptive when bubbles were released on the stigmatic surface (Zeisler 1938). To determine whether the flower is receptive during the visits by pollinators, the measurements of stigmatic receptivity were carried out in the beginning of anthesis and in the peak of visits by hummingbirds and bats (*n* = 5 flowers per time per taxon).

### Nectar volume, concentration and amount of sugars

We recorded the total amount of nectar produced (µL), concentration (%), amount of sugar (mg) and pattern of production during the anthesis in both *Ipomoea* taxa, following Galetto and Bernardello (2005). We took measurements of sugar volume using graduated microsyringes (Hamilton, NV, USA) and measurements of concentration with a handheld refractometer (0–50 %; Atago, Tokyo, Japan). The measurements were carried out in all months of flowering to include as many individuals as possible. We selected individuals according to the availability of flower buds and to avoid pseudoreplication, we excluded individuals close to each other in measurements carried out at the same time. The time and number of measurements differed according to the period of daily anthesis of each taxon: six measurements (volume, concentration and milligrams of sugar) from 0900 to 0400 hours in *I. marcellia*, and seven measurements (volume, concentration and milligrams of sugar) from 1600 to 0700 hours in *Ipomoea* aff. *marcellia.* In each period, we measured the accumulated volume of nectar in a group of previously bagged flowers that could not be accessed by flower visitors. And in each *Ipomoea* taxon, we used a group of 10–20 flowers from different individuals per measurement per time.

### Pollination experiments

To analyze the reproductive system of the studied *Ipomoea*, we carried out controlled pollination tests in the field (Radford *et al*. 1974). Frequently, the same individual was used for more than one type of pollination experiment (e.g. spontaneous self- and hand self-pollination). However, to avoid pseudoreplication, one flower per individual was used in similar experiments. Thus, flower buds of different individuals were covered with voile bags, and the following procedures were performed after the beginning of anthesis: (i) spontaneous self-pollination (*I. marcellia*, *n* = 44; *I.* aff. *marcellia*, *n* = 30)—without hand pollen transfer to the stigma; (ii) hand self-pollination (*I. marcellia*, *n* = 20; *I.* aff. *marcellia*, *n* = 15)—with hand pollen transfer to the stigma of the same flower; (iii) outcrossing intrataxa (*I. marcellia*, *n* = 19; *I.* aff. *marcellia*, *n* = 22)—with hand pollen transfer among individuals of the same taxon; and (iv) outcrossing intertaxa (*I. marcellia*, *n* = 11; *I.* aff. *marcellia*, *n* = 16)—with hand pollen transfer between taxa. After the experiments, we bagged the flowers again and monitored the production of fruits and seeds. Fruit set was measured as the number of flowers setting fruit per treatment. The seeds of the fruits were quantified for each treatment, and for the analyzes, we have used the average number of seeds per fruit.

### Pollination by hummingbirds and bats: visit number, frequency and efficiency

We determined the taxonomic identity of flower visitors, and number and frequency of visits to the flowers of both *Ipomoea* taxa through diurnal and nocturnal observations (*n* = 10 individuals per taxon). As the number of flowers can affect the visit rate, all individuals included in the analysis had from one to two open flowers. We carried out observations on different days (∼20 days), in census lasting from 30 min to 2 h, during the flowering season, in a total of 30 h for *I. marcellia* (diurnal observation = 13 h; nocturnal observation = 17 h) and 26.5 h for *Ipomoea* aff. *marcellia* (diurnal observation = 11 h; nocturnal observation = 15.5 h). Photographic records were taken to help in the identification of flower visitors and the visualization of the position of pollen placement on the animal.

We captured bats visiting *Ipomoea* flowers with mist nets (four mist nets, 7 × 2.5 m, Ecotone Inc., Spot, Poland) for later identification, record of sites of pollen deposition and the pollen load composition. The identification of pollen types found on the body of bat specimens was carried out using the reference collection of chiropterophilous plant species found in the study area (Queiroz *et al*. 2016). The pollen was removed from the body of bats with adhesive tape and examined under light microscope only in a quantitative manner, and we have registered the presence/absence of pollen from both *Ipomoea* taxa and from other plants species. The nets were opened around blooming individuals during the peak of visits by bats, between 1800 and 2100 hours. Sampling was carried out from July to October, in a total of 48 h of capture per *Ipomoea* taxon. Bats were identified in the field using field guides or consulting specialists. One or two specimens of each species were killed using a lethal chamber with ether and deposited as vouchers in the zoological collection of the Federal University of Pernambuco (UFPE).

We estimated and compared pollination services by bats and hummingbirds using as indicators of the number and frequency of flower visits and the quality of those visits. The quality of visits is measured as the number of fruits and seeds per fruit produced per flower in experiments of diurnal and nocturnal selective exposure. Each individual used in the treatment of bat and/or hummingbird pollination was considered as an experimental unit, that is, to perform the experiments (nocturnal or diurnal exposure) we used only one flower per plant individual, and a non-paired sample. Due to differences in the anthesis period, diurnal exposure of flowers varied according to taxon. In *I. marcellia* (*n* = 24 flowers) it was carried out in a single interval: 1000–1730 hours, and in *Ipomoea* aff. *marcellia* (*n* = 30 flowers) in two intervals: 1600–1730 hours and 0500–0730 hours. All flowers remained bagged except for the time of diurnal exposure. Nocturnal exposure in *I. marcellia* (*n* = 21 flowers) and *Ipomoea* aff. *marcellia* (*n* = 27 flowers) was carried out in the same interval: 1800–0430 hours. All flowers remained bagged except for the time of nocturnal exposure. We marked the flowers in both experiments and monitored fruit set and seeds per fruit set (Table 2).

### Statistical analysis

We tested for differences in floral morphometry (e.g. length and width of corolla and length of stamens and pistil) and nectar characteristics (e.g. volume, concentration and milligrams of sugar) between the two *Ipomoea* taxa with a series of *t*-tests. In the treatments of outcrossing (inter × intrataxa) and exclusion of pollinators (bat × hummingbird pollination), we compared the fruit set between treatments with a G test, taking into account that this test is more suitable for samples with expected values below five. We compared the number of seeds formed in the controlled and natural pollination treatments, and in the experiments of selective exclusion of diurnal and nocturnal visitors with Kruskal–Wallis, followed by a *post hoc* Dunn test. Whenever the statistic test so required, the data were analyzed for normality and homoscedasticity of variances. All tests followed Sokal and Rohlf (1995).

## Results

### Morphometry and flower anthesis

The flowers of *I. marcellia* had corolla, stamens and pistil significantly larger than those of *I.* aff*. marcellia* (Table 1). In addition, the edge of the corolla was more intensely folded in *I. marcellia* than in *I.* aff. *marcellia*, which allows higher exposure of anthers and stigma in the former (Fig. 1).

**Table 1.** Floral traits (mm) and nectar attributes in two co-flowering *Ipomoea* taxa (Convolvulaceae) in a Caatinga area, northeastern Brazil. *P* < 0.05 were statistically different.

Traits	*n*	*Ipomoea marcellia*	*Ipomoea* aff. *marcellia*	Comparisons
Floral tube length	30	57.52 ± 5.99	46.05 ± 3.36	*t* = **−**4.85; *P* < 0.001
Floral tube width	30	22.73 ± 3.37	18.42 ± 2.16	*t* = **−**5.05; *P* < 0.0001
Stamen length	30	55.45 ± 6.54	45.50 ± 2.63	*t* = **−**8.72; *P* < 0.0001
Pistil length	30	55.94 ± 5.78	42.33 ± 2.31	*t* = **−**10.38; *P* < 0.0001
Nectar volume (µL)	30	74.70 ± 18.88	117.40 ± 15.55	*t* = **−**8.29; *P* < 0.001
Nectar concentration (%)	30	32.95 ± 1.89	36.44 ± 2.15	*t* = **−**6.73; *P* < 0.001
Total nectar sugar (mg)	20	23.48 ± 9.44	55.90 ± 14.97	*t* = **−**8.19; *P* < 0.001

The time and total duration of anthesis differed between taxa studied. The anthesis of *I. marcellia* started in the morning, between 0900 and 1000 hours, and lasted ∼20 h. The anthesis of *Ipomoea* aff. *marcellia* started in the late afternoon, approximately at 1600 hours, and lasted ∼16 h. In both taxa, anthers were dehiscent right after bud opening and the stigma remained receptive throughout anthesis.

### Nectar volume, concentration and amount of sugar

**Figure 2.**
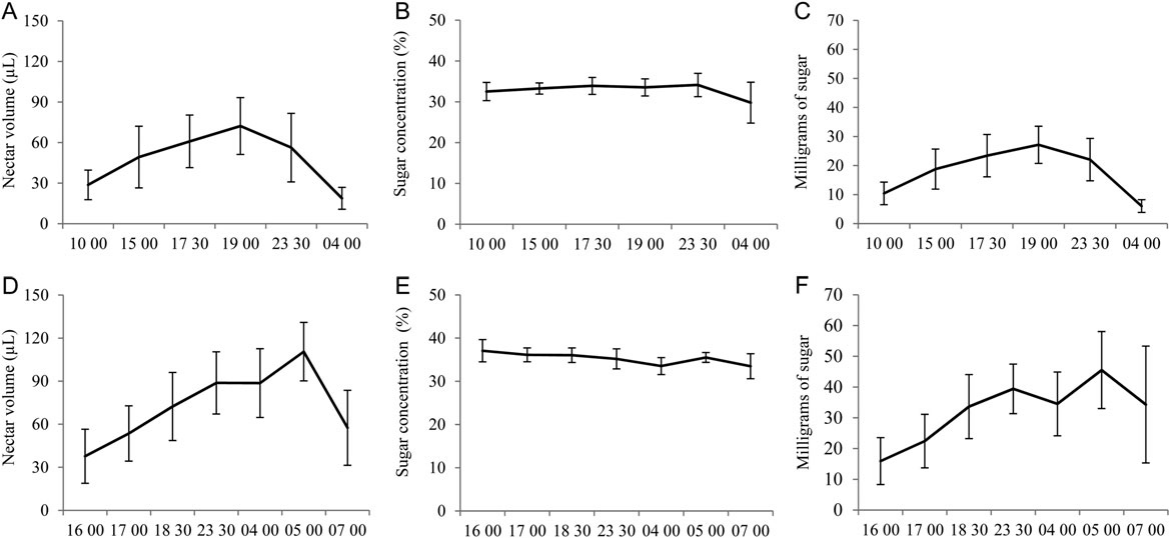
Nectar characteristics in diurnal and nocturnal periods of anthesis in flowers of two co-flowering *Ipomoea* in a Caatinga area, northeastern Brazil. (A) Volume, (B) concentration and (C) milligrams of sugar in *Ipomoea marcellia* flowers, (D) volume, (E) concentration and (F) milligrams of sugar in *Ipomoea* aff. *marcellia*. Horizontal lines join average values for each measurement time and bars indicate standard deviations. In both *Ipomoea* taxa values of *n* for volume, concentration and milligrams of sugar vary between 15 and 20 flowers per time point.

Nectar production was continuous and began with flower opening: in *I. marcellia* between 0900 and 1000 hours and in *Ipomoea* aff. *marcellia* at 1600 hours. The amount of nectar decreased only at the end of anthesis: at 0400 hours in *I. marcellia* and 0700 hours in *Ipomoea* aff. *marcellia* (Fig. 2). The volume, concentration and the total amount of sugar in the nectar of *Ipomoea* aff. *marcellia* were significantly higher than in *I. marcellia* (Table 1).

### Pollination experiments

Both *Ipomoea* taxa were obligate xenogamous (sensu Cruden 1977): we observed fruit and seed set exclusively in the treatment of hand cross-pollination (Table 2). However, in spite of higher fruit and seed set in outcrossing intrataxa than outcrossing intertaxa in *I. marcellia* (*Z* = 1.10; gl = 1; *P* > 0.05) and in *I.* aff. *marcellia* (*Z* = 2.14; gl = 1; *P* > 0.05), these differences were not statistically significant. Natural seed set in both *Ipomoea* taxa did not differ from outcrossing treatments (Table 2).

**Table 2.** Fruit and seed set in two co-flowering *Ipomoea* taxa (Convolvulaceae) under different pollination conditions, in a Caatinga area, northeastern Brazil. Fruit set**:** (i) Outcrossing—values within the same column followed by the same letter were not statistically different (*P* > 0.05) and (ii) animal pollination—values within the same column followed by the same subscribed number were not statistically different (*P* > 0.05); Seed set: (iii) Outcrossing and pollination natural—values within the same column followed by the same subscribed letter were not statistically different (*P* > 0.05); (iv) Natural, bat and hummingbird pollination—values within the same column followed by the same subscribed number were not statistically different (*P* > 0.05) NA, not available data; *Mean ± SD; **Flowers used as receptors of interspecific pollen.

Treatments	*Ipomoea marcellia*			*Ipomoea* aff. *marcellia*		
	*n*	Fruits (%)	Seeds per fruit*	*n*	Fruits (%)	Seeds per fruit*
Spontaneous self-pollination	44	0 (0)	0 (0)	30	0 (0)	0 (0)
Hand self-pollination	20	0 (0)	0 (0)	15	0 (0)	0 (0)
Outcrossing (intrataxa)	19	12 (63.15)^a^	2.72 ± 1.19^a^	22	6 (27.27)^a^	3.5 ± 0.54^a^
Outcrossing (intertaxa)	11**	4 (36.36)^a^	2.66 ± 1.63^a^	16**	2 (12.5)^a^	2.0 ± 1.41^a^
Natural pollination	15	NA	2.88 ± 0.76^a,1,2^	15	NA	3.26 ± 0.45^a,1,2^
Bat pollinated	21	2 (9.52)^1^	2.00 ± 0.0^1^	27	12 (44.44)^1^	3.77 ± 0.44^1^
Hummingbird pollinated	24	7 (33.33)^1^	3.57 ± 0.78^2^	30	4 (18.18)^2^	2.50 ± 0.57^2^

### Pollination by hummingbirds and bats: visit number, frequency and efficiency

**Figure 3.**
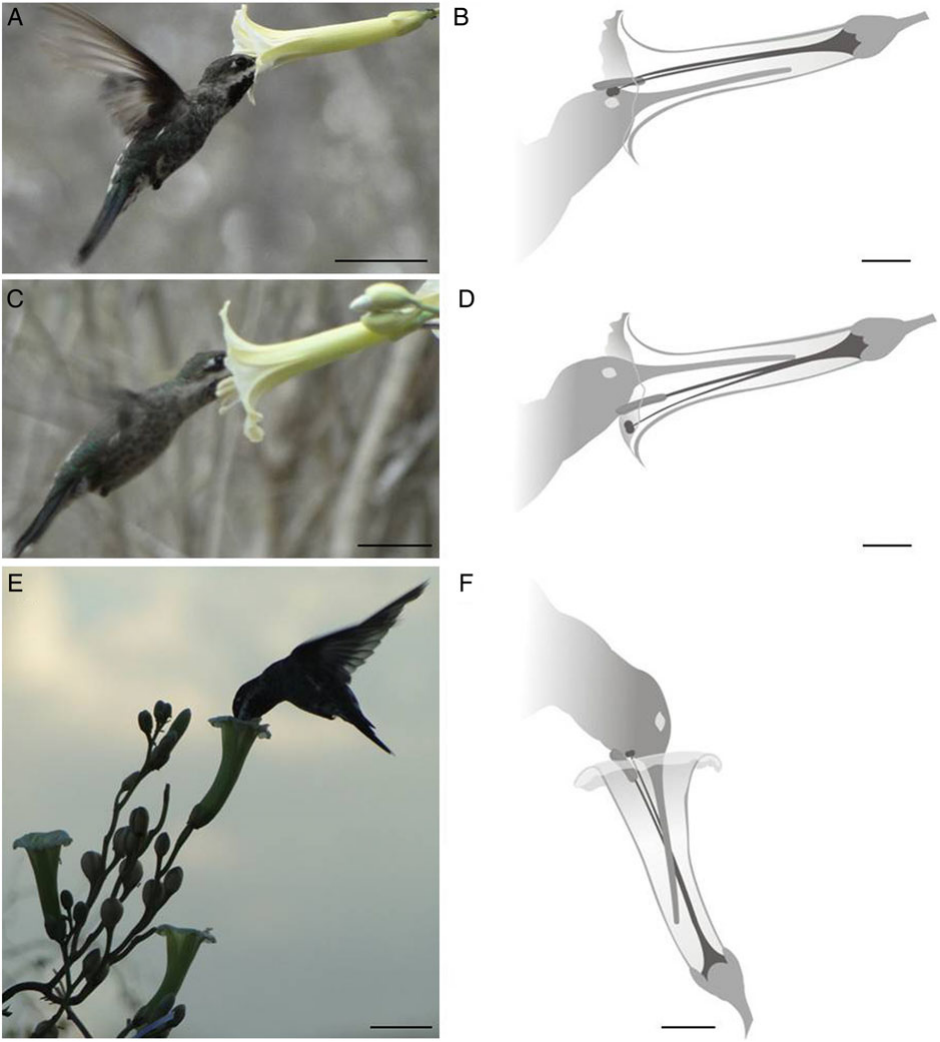
Pollination by hummingbirds in two co-flowering *Ipomoea* in a Caatinga area, northeastern Brazil. *Heliomaster squamosus* accessing the horizontally arranged flower of *I. marcellia*, with nototribic (A and B) and sternotribic pollen deposition (C and D), and accessing *Ipomoea* aff. *marcellia* flower, with exclusively sternotribic pollen deposition (E and F). Scale bars: A, C and E = 3 cm, B, D and F = 1 cm.

**Figure 4.**
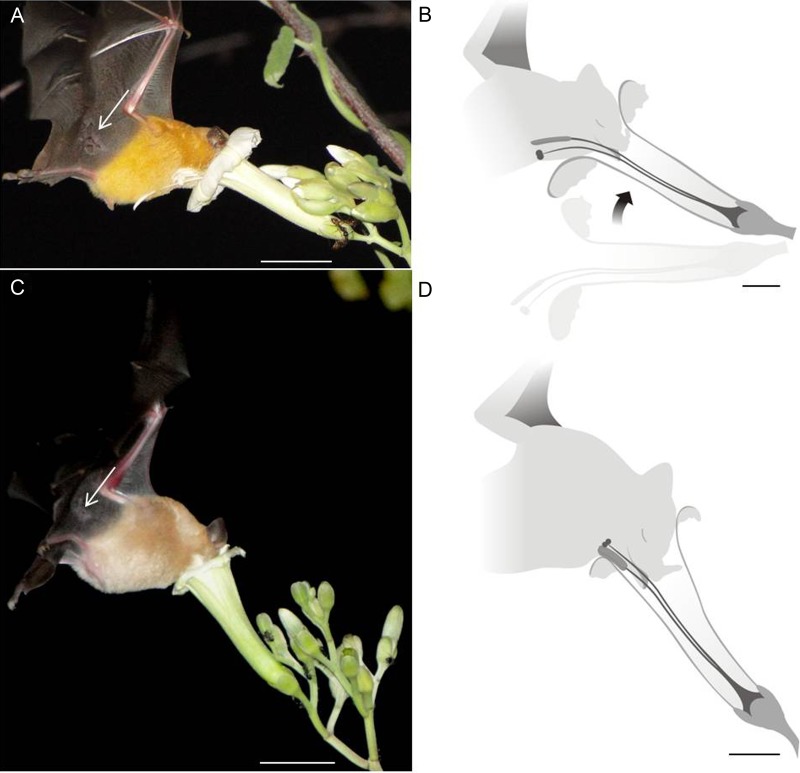
Pollination by glossophagine bats in two co-flowering *Ipomoea* in a Caatinga area, northeastern Brazil. Glossophagine bats accessing a flower of *I. marcellia* with sternotribic pollen deposition on the neck and breast (A and B); and accessing the vertically arranged flower of *Ipomoea* aff. *marcellia* with sternotribic pollen deposition exclusively on the neck (C and D). White arrows show individual marks on the bat's patagium recorded at different nights on flowers of close individuals. This is evidence that the same individual bat can access flowers of both *Ipomoea* taxa. Scale bars: A and C = 2 cm, B and D = 1 cm.

The two *Ipomoea* taxa shared the same diurnal and nocturnal pollinator species (Figs 3 and 4). *Heliomaster squamosus* (Family Trochilidae) was the only diurnal visitor recorded (Fig. 3). This hummingbird accessed the flowers of *I. marcellia* during a long daily period (0900–1730 hours). We recorded a total of 42 visits (1.90 visits h^−1^). In those visits, the pollen was deposited on the head (Fig. 3A and B) and neck of the hummingbird (Fig. 3C and D). The visits of *H. squamosus* to *Ipomoea* aff. *marcellia* occurred at two intervals (1700–1730 hours and 0530–0730 hours). We recorded 11 visits during the whole-observation time (0.57 visits h^−1^), in which the pollen was exclusively deposited on the neck of the hummingbird (Fig. 3E and F).

The main nocturnal visitors were the bats *Glossophaga soricina* and *Lonchophylla mordax* (Glossophaginae). Hawkmoth visits were occasional; we recorded one visit of *Agrius cingulata* to both *Ipomoea* taxa*.* Bat visits to flowers were concentrated between 1800 and 2000 hours*.* We recorded 11 visits (0.57 visits h^−1^) to *I. marcellia*, in which the pollen was deposited in the region between the neck and breast of the bat (Fig. 4A and B). We recorded 12 visits (0.64 visits h^−1^) of these bats to *Ipomoea* aff. *marcellia,* in which the pollen was deposited on the chin and neck of the bats (Fig. 4C and D). We collected 13 bat specimens with pollen of *Ipomoea* taxa, *L. mordax* (eight individuals) and *G. soricina* (five individuals). The pollen load found in 100 % of the bats analyzed was a mixture of pollen of *Ipomoea* taxa with pollen of other plants that overlap part of their flowering period. Among those plants were *Pilosocereus chrysostele* (Cactaceae), *Pseudobombax marginatum* (Bombacaceae) and *Encholirium spectabile* (Bromeliaceae).

In *Ipomoea* aff. *marcellia*, pollination by bats resulted in more fruits produced than in hummingbird pollination (*G* = 5.47; df = 1; *P* = 0.01). The average number of seeds per fruit in *I.* aff. *marcellia* in natural pollination did not differ of bat (*Z* = 1.80; df = 1; *P* < 0.05) or hummingbird pollination (Z = 1.91; df = 1; *P* < 0.05). However, seed set was higher in bat pollination when compared with hummingbird pollination in this taxon (*Z* = 3.19; df = 1; *P* < 0.05). Although we did not observe significant differences among bat and hummingbird pollination in terms of fruits set in *I. marcellia* (*G* = 2.53; df = 1; *P* > 0.05), in terms of seed set, hummingbird pollination was more effective and significantly different from bat pollination (*Z* = 3.00; df = 1; *P* < 0.05). In addition, the number of seeds per fruit in *I. marcellia* in the treatment of natural pollination did not differ from that seen in bat (*Z* = 2.07; df = 1; *P* > 0.05) or hummingbird pollination (*Z* = 1.59; df = 1; *P* > 0.05).

## Discussion

In the present work, we have described two new cases of shared pollination by vertebrates (bat and hummingbird) in *Ipomoea*. Pollination by vertebrates has been reported for the genus (see McDonald *et al*. 2011). However, *Ipomoea* consists of a predominantly insect pollinated group, mainly by bees (Galetto *et al*. 2002; Galetto and Bernardello 2004; McDonald *et al*. 2011 and references there in). Although bat pollination is suggested for *I. albivenia*, *I. ampullacea*, *I. arborescens* and *I. neei* (Dobat and Peikert-Holle 1985; Fleming *et al*. 2009), it has been rarely confirmed by direct observations (Sánchez and Medellín 2007; Caballero-Martínez *et al*. 2012). Vertebrate pollination in *I. murucoides*, a primarily chiropterophilous species (Butanda-Cervera *et al*. 1978), was the only case in which diurnal versus nocturnal pollinator efficiency in *Ipomoea* has been previously tested (Caballero-Martínez *et al*. 2012).

If pollination systems are conceptualized as a continuum, we would have at one end very specialized systems, such as in long tubular flowers of *Centropogon nigricans* pollinated by a single species of bat (Muchhala 2006*b*), and in another end, generalist systems with plant species and its numerous floral visitors (Johnson and Steiner 2000; Fenster *et al*. 2004; Maldonado *et al*. 2013; Avila *et al*. 2015). In this continuum, our bat–hummingbird pollination system would be closer to those more specialized. Some bat–hummingbird pollination systems have been considered as generalized (Muchhala *et al*. 2009) or intermediate between ornithophily and chiropterophily (Sazima *et al*. 1994). We support the latter view, since the co-flowering *Ipomoea* taxa had, in general, a set of floral traits (e.g. cup-shaped, whitish green and accessible flowers, anthesis and nectar by day and night) that favours pollination by only two groups of vertebrates, bats and hummingbirds.

The whitish green colour of the flowers in the two *Ipomoea* taxa is not a typically ornithophilous colour, but more associated with chiropterophily (Faegri and van der Pijl 1979; Tschapka and Dressler 2002; Willmer 2011). However, similarly to what was observed in *Burmeistera* flowers (Muchhala 2006*a*), it does not hinder visits by hummingbirds. Another factor, day- and night-time availability of nectar, also favours bat–hummingbird pollination (Sazima *et al*. 1994; Muchhala *et al*. 2009; Christianini *et al*. 2013). This kind of pollination system has a high energy cost, because both bats and hummingbirds show high metabolic rates, due to their sizes and hovering flight (Nicolson and Fleming 2003; Tschapka 2004). Indeed, the nectar concentration of the two studied *Ipomoea* was higher than that described for chiropterophilous plants (2–29 %; von Helversen 1993; Fleming *et al*. 2009) and ornithophilous flowers (15–25 %; Hainsworth and Wolf 1972; Baker 1975; Baker and Baker 1983; Proctor *et al*. 1996).

In addition, the period of day–night anthesis of studied *Ipomoea* taxa is also another factor favouring both bat and hummingbirds activities. Plant species with prolonged anthesis (e.g. >24 h) are usually visited by diurnal and nocturnal animals (Sazima *et al*. 1994; Muchhala *et al*. 2009; Caballero-Martínez *et al*. 2012; Amorim *et al*. 2013; Christianini *et al*. 2013; Aguilar-Rodríguez *et al*. 2015). In such cases, the duration (Willmer 2011) and the beginning of anthesis (Aguilar-Rodríguez *et al*. 2015) are important floral attributes that can provide clues about the contribution, in terms of fruit and seed set, of the different groups of pollinators (nocturnal and diurnal). Thus, the differences in the beginning and in the total duration of anthesis between the two *Ipomoea* taxa can affect the interaction with their pollinators, favouring hummingbirds in *I. marcellia* (daytime onset, duration: 20 h) and bats in *I*. aff. *marcellia* (twilight beginning; duration: 16 h).

In general, in species of plants using nocturnal and diurnal pollinator services, complementarity between these two groups of animals is noted in the pollination of these plants (Muchhala *et al*. 2009; Amorim *et al*. 2013; Aguilar-Rodríguez *et al*. 2015; Cruz-Neto *et al*. 2015). Negative effect has been rarely observed in terms of fruit and seed set, as reported to *Inga* (Avila *et al*. 2015). In the present study, bats and hummingbirds played complementary roles in the pollination of both *Ipomoea*. However, such roles diverged among the two taxa. Although hummingbirds presented higher frequency of visits (quantity component) than bats, both seem to contribute equally to a successful fruit set in *I. marcellia*. However, the higher amount of seeds per fruit resulting from the pollination by hummingbirds in this plant can result from the longer duration of the diurnal anthesis, which favours hummingbird visit rate. However, the frequency of visits should not be so relevant to determine the importance of the pollinator in *I.* aff. *marcellia*, as bats and hummingbirds showed the same visit rates in this plant, but bat pollination was significantly more successful, both in fruit and seed set.

When plant species share a flowering period and have the same pollinators, loss of pollen, due to mixing with foreign pollen, is a common condition (Murcia and Feinsinger 1996; Muchhala and Jarrin-V 2002). However, some factors may contribute to minimize pollen loss, such as flowering period displacement (Heithaus *et al*. 1975) or specialization on different pollination strategies (Christianini *et al*. 2013). The second factor was more important in the studied *Ipomoea*, since intertaxa differences in the morphometry and spatial orientation of flowers allow to use slightly different parts of the pollinator's body for pollen deposition. The floral morphology plays an important role in reducing competition for pollinators (Dressler 1968; Tschapka *et al*. 2006; Muchhala 2008; Armbruster *et al*. 2014) and favour reproductive isolation among sympatric taxa (Muchhala 2003; Muchhala and Potts 2007; Christianini *et al*. 2013; Armbruster *et al*. 2014). Similarly to *Burmeistera*, which are also pollinated by bats and hummingbirds (Muchhala 2003), divergences in floral morphology in *Ipomoea* played a central role in pollinator specialization and made possible the co-occurrence and pollinator sharing in these taxa.

Despite the self-incompatibility observed in both *Ipomoea*, we observed fruit and seed production by intertaxa outcrossing. Hybridization under natural conditions is very rare in *Ipomoea* due to cross-incompatibility (Cao *et al*. 2009). However, under controlled conditions some *Ipomoea* species of economic interest have been able to generate hybrids (Abel and Austin 1981; Cao *et al*. 2009). When congeneric species have contact with each other, hybridization may not occur due to the existence of pre- and post-mating barriers (Templeton 1989). Temporal displacement of flowering peaks, strong pollinator specificity and high flower constancy in the shared pollinators can contribute to restrict hybridization (Marques *et al*. 2007). However, we did not observe any of these barriers in the two studied *Ipomoea.* We believe, though, that two other barriers can negatively affect the success of intertaxa outcrossing by limiting the formation of hybrids under natural conditions in these two taxa: differences in the floral morphometry and self-incompatibility.

In the case of the studied *Ipomoea*, two main factors may make a bat–hummingbird pollination system advantageous. The first factor is related to the quality of the pollen deposited on the stigma of *Ipomoea* flowers. Due to their high energy requirements, bats and hummingbirds need to visit several flowers, and, therefore, can show mixed pollen load (Murcia and Feinsinger 1996; Muchhala and Jarrin-V 2002). During part of the flowering season of *Ipomoea* taxa, other chiropterophilous and ornithophilous plants were also in blossom (Quirino and Machado 2014). Those plants deposit pollen on the same sites of the pollinator's bodies where both *Ipomoea* did. Indeed, all bats collected in Farm Almas had pollen from other chiropterophilous plants (J. Queiroz, unpubl. data). Although we did not capture hummingbirds during fieldwork, we observed *H. squamosus* visiting flowers of *Melocactus* sp., which probably led to mixed pollen too. This mixed pollen pattern can have negative implications to pollination, by decreasing pollinator effectiveness and consequently favouring generalization in detriment of specialization (Muchhala and Jarrin-V 2002; Muchhala *et al*. 2009).

A second factor is related to an apparent advantage of bat–hummingbird pollination systems in environments where pollinator populations can undergo seasonal fluctuations (Waser *et al*. 1996) or be small (Wolff *et al*. 2003). In the Caatinga, where rainfall changes largely between years (Prado 2003), pollinator populations can undergo seasonal variations and be less abundant during droughts (Aguiar and Martins 1997; Duarte Junior and Schlindwein 2005). As the *Ipomoea* taxa studied here bloom in the dry season, trusting the pollination service to a single group of pollinators can be risky. When specialization in a particular pollinator group results in ecological dependence, the risk of extinction is higher (Johnson 2010). Hence, in both cases (pollinator ‘infidelity’ and fluctuations in pollinator populations) the natural selection favour shared pollination systems.

## Conclusions

Both studied *Ipomoea* taxa blossom in the same period of the year, occur simultaneously in mixed vegetation patches, share the same vertebrate pollinators and could probably hybridize. However, we consider that some factors should minimize pollen exchange among those taxa and, consequently, favour their reproductive isolation, namely, variations in flower morphology and morphometry, floral orientation, anthesis time and pollen deposition on distinct places of the pollinator body, both in bats (*I. marcellia*: breast/*I.* aff. *marcellia*: chin and throat) and hummingbirds (*I. marcellia*: throat and forehead/*I.* aff. *marcellia*: chin). Although we classified the two studied *Ipomoea* in a bat–hummingbird pollination system, those plants seem to have different degrees of specialization: the floral traits of *I.* aff. *marcellia* are more consistent with chiropterophily, which should explain the higher effectiveness of bats in the pollination of this taxon, and most attributes of *I. marcellia*, as well as its larger flower size and longer anthesis, do not seem to restrict either hummingbirds or bats. Hence, *I.* aff. *marcellia* can be seen as comparatively more specialist than *I. marcellia*.

## Sources of Funding

The work was supported by PELD-CNPq (Long-Term Ecological Program) and by FACEPE (APQ-1096-2.03/08).

## Contributions by the Authors

The authors contributed equally in all activities of this work, its conception, data collection and the writing of the manuscript.

## Conflict of Interest Statement

None declared.
